# Myositis-associated antibodies predict the severity of lung involvement in adult patients with inflammatory myositis − a cohort study of 70 adult patients with myositis in a single center

**DOI:** 10.3389/fmed.2024.1340310

**Published:** 2024-03-28

**Authors:** Josefin Marklund, Balsam Hanna, Tao Jin, Rille Pullerits

**Affiliations:** ^1^Department of Rheumatology, Sahlgrenska University Hospital, Gothenburg, Sweden; ^2^Department of Rheumatology and Inflammation Research, Institute of Medicine, Sahlgrenska Academy at University of Gothenburg, Gothenburg, Sweden; ^3^Department of Clinical Immunology and Transfusion Medicine, Sahlgrenska University Hospital, Gothenburg, Sweden

**Keywords:** idiopathic inflammatory myopathy, myositis-associated antibody, myositis-specific antibody, anti-Ro/SSA52, lung involvement

## Abstract

**Introduction:**

Idiopathic inflammatory myopathies (IIMs) encompass a diverse group of diseases characterized by considerable variability in clinical manifestations, antibody profiles, and responsiveness to immunosuppressive therapies. This study aimed to investigate the association between organ involvement and distinct myositis autoantibodies in individuals with IIM in a single-center cohort.

**Methods:**

Patients with ICD diagnoses M33.1, M33.2, M33.9, or M609 who (1) had been tested with Euroline blot assay for myositis autoantibodies and (2) met the classification criteria of definite/probable polymyositis (PM) or dermatomyositis (DM), anti-synthetase syndrome (ASS), or inclusion body myositis (IBM) were included. Medical journals were retrospectively examined with respect to clinical disease features.

**Results:**

Seventy patients (median age 58 years; 66% females) were included and represented the following diagnosis: PM (*n* = 23), DM (*n* = 21), ASS (*n* = 23), and IBM (*n* = 3). Most of the patients (87%) presented a muscle biopsy indicative of myositis. The presence of autoantibodies was as follows: myositis-specific antibodies, MSA (*n* = 53), myositis-associated antibodies, MAA (*n* = 33), both MSA + MAA (*n* = 24), MSA only (*n* = 29), MAA only (*n* = 9), no MSA, or MAA (*n* = 8). Anti-Jo-1 was the most common MSA (19%), whereas the most common MAA was anti-Ro/SSA52 (31%). We observed a significant association between antibody patterns and lung disease. In our cohort, 47% of the patients in the whole study group, 86% of patients with anti-SSA52, and 100% with anti-Jo-1 had pulmonary involvement. Patients with both MSA and MAA had a higher incidence of lung disease and decreased CO-diffusion capacity. This was especially prominent in anti-Ro/SSA52-positive patients. Interestingly, none of the patients suffered from lung disease if only antibodies against Mi-2α, Mi-2β, NXP2, HMGCR, and TIF1γ were present or no MSA/MAA were detected.

**Discussion:**

The simultaneous presence of both MAA and MSA indicates an increased risk of lung involvement in patients with inflammatory myopathies. The presence of any MAA, and especially anti-Ro/SSA52, is associated with more severe pulmonary disease. Our data suggest that MAA antibodies might be relevant markers for early detection and treatment of lung involvement in IIM.

## 1 Introduction

The idiopathic inflammatory myopathies (IIMs) constitute a diverse and rare group of systemic disorders characterized by muscle weakness and inflammatory infiltrates within skeletal muscles. Common hallmarks of IIM encompass progressive muscle weakness, elevated muscle enzyme levels, signs of inflammation in muscle biopsy or magnetic resonance imaging, and myopathic findings in electromyography (EMG) studies (1). According to the clinical features, antibody profile, pathological pattern found in the muscle biopsy, and responsiveness to the immunosuppressive treatment, myositis in adult patients is subdivided into different entities such as polymyositis (PM), dermatomyositis (DM), and inclusion body myositis (IBM) (1). Notably, patients with DM also present characteristic skin manifestations. Recent developments in identifying new specificities of myositis-specific autoantibodies (MSAs), myositis-associated antibodies (MAAs), and the growing understanding of associated clinical features have led to the recognition of additional subsets of inflammatory myopathies, including anti-synthetase syndrome (ASS), immune-mediated necrotizing myopathy (IMNM), clinically amyopathic dermatomyositis (CADM), and myositis associated with overlap syndromes and cancer (2).

The purpose of this study was to explore the clinical and laboratory manifestations in a group of patients with a confirmed myositis diagnosis in a single center. Given that pulmonary involvement represents the most frequently observed severe organ manifestation in myositis, our focus was to identify clinical patterns that could be associated with susceptibility to lung involvement and, thus, to identify the patients in need of intensified treatment strategies.

## 2 Materials and methods

### 2.1 Patients

**Figure 1:**
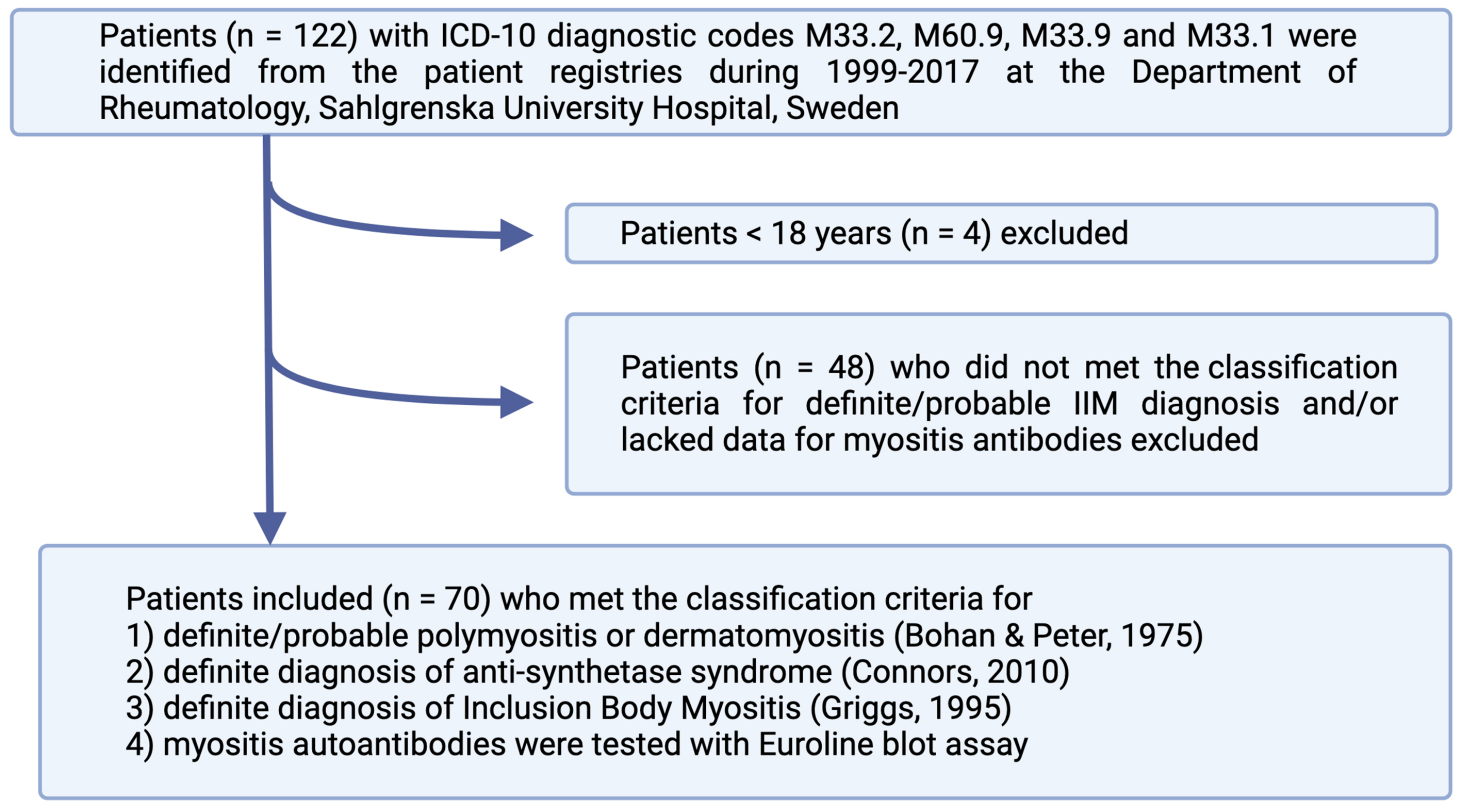
Flowchart of patient inclusion in the study. The patients were identified from patients’ registries according to ICD-10 codes and included if they fulfilled the classification criteria for either definite or probable IIM. IIM, idiopathic inflammatory myopathies.

We identified all patients with the following ICD-10 diagnostic codes: M33.2 (PM), M60.9 (myositis, unspecified), M33.9 (dermato-PM, unspecified), or M33.1 (other, DM) in the medical journal database at the Rheumatology Department of Sahlgrenska University Hospital, Gothenburg, Sweden, from 1999 to 2017. Altogether, 122 patients were identified. The patients fulfilling the following inclusion criteria were included: (1) adult patients above 18 years of age; (2) met the classification criteria of either definite or probable PM or DM according to Bohan and Peter criteria (3, 4); definite diagnosis of ASS according to Connors et al. (5) or definite diagnosis of IBM according to Griggs et al. (6); (3) blood samples had been tested with Euroline blot assay (Euroimmun, Germany) for myositis autoantibodies. The following patients were excluded: 4 patients <18 years, 1 patient who was lost for follow-up at the Rheumatology Clinic, and 48 patients who did not meet the classification criteria for a definite/probable diagnosis and/or lacked the data regarding myositis antibodies (Figure 1). In total, 70 patients were included in the study group. The baseline characteristics of the patients are summarized in Table 1.

**Table 1:** Demographic and disease-related characteristics of 70 patients with idiopathic inflammatory myopathies.

Age at diagnosis (years), median (IQR)	58 (38–73)
Gender, females, *n* (%)	46 (66)
Disease duration at assessment (months), median (IQR)	6 (2–12)
Total follow-up time (months), median (IQR)	18 (10–48)
IIM subtype according to classification criteria, *n* (%)	
Inclusion Body Myositis	3 (4%)
Polymyositis	23 (33%)
Dermatomyositis	21 (30%)
Anti-synthetase syndrome	23 (33%)
Muscle biopsy, *n* (%)	
performed	64 (87%)
histopathology suggestive of myositis	61 (95%)
Autoantibody profile, *n* (%)	
Myositis-specific antibody (MSA) (including anti-HMGCR)	53 (75%)
Myositis-associated antibody (MAA) (including anti-cN1A)	33 (47%)
Presence of both MSA and MAA	24 (34%)
Seronegative (no presence of MSA, MAA)	8 (11%)
Presence of clinical features*, *n* (%)	
Muscle symptoms	58 (83%)
Fever	15 (21%)
Fatigue	40 (57%)
Weight loss	27 (38%)
Raynaud syndrome	24 (34%)
Arthralgia/arthritis	37 (53%)
Calcinosis	2 (3%)
Mechanic’s hands	17 (24%)
Pulmonary involvement /Interstitial lung disease	33 (47%)
Dysphagia/esophagus involvement	24 (34%)
Carpal tunnel syndrome**	12 (17%)
Various skin manifestations	30 (43%)
Malignancy (diagnosed up to 3 years from IIM onset)	9 (13%)
Biopsy verified heart involvement	1 (1%)

IQR, interquartile range; IIM, idiopathic inflammatory myopathy; anti-HMGCR, antibodies against 3-hydroxy-3-methylglutaryl coenzyme A.

*The clinical feature was considered missing if it was not documented/diagnosed.

**Includes only carpal tunnel syndrome verified by nerve conduction investigation.

In 12 patients, a rheumatic disease had been diagnosed before the onset of IIM. The diagnoses comprised SLE (*n* = 1), psoriatic arthritis (PsA) (*n* = 2), systemic sclerosis (SSc) (*n* = 1), rheumatoid arthritis (RA) (*n* = 2), primary Sjögren’s syndrome (pSS) (*n* = 2), sarcoidosis (*n* = 1), and unspecified poly- or oligoarthritis (*n* = 3). Two patients died during the follow-up period.

### 2.2 Clinical and laboratory assessment

The medical records of patients were carefully reviewed, and information regarding clinical, laboratory, and disease-related parameters, organ involvement, as well as medication, was collected. The retrospective study data collection covered the period from diagnosis until the end of 2017.

The involvement of different organs was defined as follows:

The patient was considered to have lung involvement if the following changes were described in a radiological (HRCT) examination: ground glass opacities, pulmonary fibrosis, changes characteristic of usual interstitial pneumonia (UIP), non-specific interstitial pneumonia (NSIP), or cryptogenic organizing pneumonia (COP). Heterogenous parenchymal radiological changes (basilar infiltrates) in HRCT, together with symptoms of dyspnea and/or decreased pulmonary function tests with decreased CO-diffusion capacity, were also considered as lung involvement. This assessment excluded enlarged lymph nodes, serositis, bronchiectasis, and pulmonary infiltrates typical for infectious pneumonia that resolved after antibiotic treatment.

Involvement of the heart due to IIM was considered if a supporting heart biopsy was present or a magnetic resonance tomography (MRT) investigation showed “delayed enhancement” and/or clearly described myocarditis together with supportive clinical symptoms.

Skin rash compatible with DM was defined if the following was identified in the patient medical records: Gottron’s sign or papules, heliotrope exanthema, Holster sign, typical periungual redness, characteristic rash at chest, back, or on extremities. If skin changes were unspecific but a skin biopsy showed histopathological features compatible with DM, the rash was defined as DM-specific. The following skin changes were excluded: unspecific redness on extremities, livedo reticularis, eczema, acne, and erythema nodosum.

Mechanical hands were defined either as such mentioned by the treating rheumatologist or by a description compatible with this condition.

The IIM was defined as cancer-associated myositis (CAM) when the IIM patient received a cancer diagnosis within a 3-year period after the IIM diagnosis, or alternatively, the patient with diagnosed cancer developed within a 3-year period an inflammatory myopathy fulfilling the IIM classification criteria.

An electromyographic (EMG) pattern was considered indicative of IIM if the following changes were registered: abnormal increased spontaneous activity such as fibrillations in needle positions (even at rest), high-frequency recurrent discharges, positive sharp waves; changes in motor unit potentials (MUPs) such as polyphasic, low amplitude, and/or short duration MUP.

The muscle biopsy was considered positive for IIM if the final assessment by the pathologist stated that the muscle biopsy findings were consistent with or supportive of either IBM (*n* = 3), DM (*n* = 19), IMNM (*n* = 9), or inflammatory myopathy/myositis (*n* = 30). Histopathological features defined as typical for IBM were inflammatory cellular infiltrates invading muscle fibers and located predominantly at the endomysial area, muscle fiber atrophy, and vacuolated muscle fibers with rimmed vacuoles (P62-positive inclusion bodies). Histopathology features considered indicative of IMNM were the presence of prominent muscle fiber necrosis together with myofiber regeneration (numerous small fibers expressing fetal and embryonic myosin), scarcity of inflammatory cell infiltration (if present, mainly CD68-positive macrophages, no CD3-positive cells), and deposition of the complement membrane attack complex C5b-9 (MAC) in necrotic muscle fibers and in connection to vessels. Histopathological features considered indicative of DM were perifascicular degeneration and atrophy of muscle fibers, the presence of perivascular and/or perimysial inflammatory infiltrates (B- and T-cells, CD68-positive cells), upregulation of MHC-I, and positive staining of MAC in vessels adjacent, especially to the periphery of the fascicles. The patient was considered to have inflammatory myopathy/myositis if the characteristic histopathological features for DM, IBM, and IMNM as described above were lacking, but changes indicative of myopathy were still evident in the muscle biopsy: perimysial, endomysial, and/or perivascular inflammatory infiltrates, scattered necrotic muscle fibers, regenerating myofibers with the presence of central nuclei, and MHC-I upregulation. Involvement of the esophagus was deemed if pathological findings were recorded at an esophagus manometry investigation or dysphagia and swallowing difficulties were clearly documented in medical records.

The diagnosis of carpal tunnel syndrome (CTS) was considered if clinical symptoms unilaterally or bilaterally were present, and the finding was confirmed by an electroneurography investigation.

### 2.3 Myositis-specific and myositis-associated antibodies

The autoantibodies were analyzed if requested by clinicians as part of the IIM investigation. The autoantibodies were categorized either as myositis-specific antibodies (MSAs – against Jo-1, Mi-2α, Mi-2β, SRP, OJ, EJ, SAE, PL-7, PL-12, TIF1γ, MDA-5, and NXP2) or MAA (against PM/Scl-75, PM/Scl-100, Ku, SSA52, SSA60, and RNP).

Screening for antinuclear antibody (ANA) specificities was performed with the automatic multiplex method (BioPlex® 2,200 System, Bio-Rad, Hercules, CA, USA) according to clinical routine care at the accredited Laboratory of Clinical Immunology, Sahlgrenska University Hospital, Gothenburg. All positive ANA-specificities were thereafter confirmed with another method. The following confirmation methods were used: Crithidia luciliae test for anti-dsDNA (ImmunoConcept, Sacramento, CA), automated ELISA-based test system Alegria® (Orgentec Diagnostics, Mainz, Germany) for anti-SSA52, and line blot ANA Profile 5 IgG for all other ANA-specificities (Euroimmun, Lübeck, Germany) according to the manufacturer’s recommendations.

The commercial myositis line blot assay (EUROLINE Autoimmune Inflammatory Myopathies 16 Ag (IgG) Profile, Euroimmun AG, Lübeck, Germany) was used and consisted of a membrane strip coated with 16 autoantigens, such as Mi-2α, Mi-2β, TIF1γ, MDA-5, NXP2, SAE1, Ku, PM-Scl-100, PM-Scl-75, Jo-1, SRP, PL-7, PL-12, EJ, OJ, and SSA/Ro-52. The procedure was carried out using a fully automated EUROBlotOne device (Euroimmun AG, Lübeck, Germany) according to the manufacturer’s instructions at the accredited Laboratory of Clinical Immunology, Sahlgrenska University Hospital. The band intensity was evaluated by the EUROLineScan program. According to the manufacturer, the band intensity thresholds of 7–14 correspond to borderline values, 15–35 to low positive (+), 30–70 to moderately positive (++), and > 70 to strongly positive (+++). Results that were borderline, according to this system, were considered negative.

The data regarding additional MAA anti-cN1A (antibodies against cytosolic 5′-nucleotidase 1A) and MSA anti-HMGCR (antibodies of the IgG subclass against 3-hydroxy-3-methylglutaryl coenzyme A) were also included when available and had been requested by clinicians due to the clinical suspicion of IBM or IMNM. The analyses were performed according to the manufacturer’s instructions at the accredited Laboratory of Clinical Immunology, Sahlgrenska University Hospital. Anti-cN-1A was measured using a commercially available ELISA kit (Euroimmun AG, Lübeck, Germany) on three occasions, and a ratio ≥ 1 was considered positive. Anti-HMGCR was measured using a QUANTA Lite® HMGCR ELISA assay (Inova Diagnostics, Inc., San Diego, CA, USA) in 12 patients. The values of ≥20 units were considered positive.

### 2.4 Treatment

Detailed information regarding the current treatment with glucocorticoids (GCs), disease-modifying antirheumatic drugs (DMARDs), and biological drugs was obtained from medical records. To estimate the need for immunosuppression during the disease course (as a surrogate marker for overall disease severity for each patient), we classified the drugs according to a score system as previously described (7) and calculated a total value for each patient. The immunosuppression, if designated for treatment with IIM, was graded as follows: 0 point – no treatment; 1 point – conventional DMARDs such as azathioprine, methotrexate, and mycophenolate mofetil; 2 points – treatment period with intravenous immunoglobulins; 3 points – treatment period with rituximab, cyclophosphamide (CYC), abatacept, or plasmapheresis. All GCs were converted according to the “Steroid Conversion Calculator” to the equivalent dose of prednisolone, and the accumulated GC dose was calculated.

### 2.5 Statistical analysis

Statistical analysis was performed using GraphPad software version 9.0 (GraphPad Prism, San Diego, USA). The following non-parametric statistical tests were used, if appropriate: Kruskal–Wallis’s test, two-tailed Mann–Whitney *U*-test, and two-tailed Spearman rank correlation test (as described in figure legends, GraphPad Prism). Fisher’s exact probability test was used to assess differences between groups regarding disease characteristics. All continuous values are expressed as the median and 25^th^–75^th^ percentiles. A *p*-value of <0.05 was regarded as being statistically significant.

## 3 Results

### 3.1 Patient characteristics

The demographic and disease-related variables of the IIM patient cohort are shown in Table 1. Seventy patients met the study inclusion criteria and comprised the following diagnoses according to the IIM classification criteria as specified above: 33% PM, 30% DM, 33% ASS, and 4% IBM. The median age at diagnosis was 58 years, and 66% of the patients were females. Patients diagnosed with ASS were younger (median age 47 years), whereas patients fulfilling the IBM diagnosis classification were significantly older (median age 80 years).

### 3.2 Clinical features of IIM

The occurrence of various clinical disease features is presented in Table 1.

The muscle biopsy was performed in 87% of patients (64 of 70), and 95% of them (61 of 64) presented histopathological features indicative of myopathy. Additionally, a skin biopsy consistent with DM was presented in 13 cases. An EMG pattern consistent with inflammatory myopathy was registered in 70% (44 of 63) patients.

The most common affected organ, following muscles, skin, and joints, was the lungs; 47% of the patients had lung involvement. In one patient, the involvement of the heart was clinically suspected and the biopsy was verified. Of note, in 17% of the cohort (*n* = 12), nerve conduction studies showed bilateral or unilateral changes, as in CTS. In 7 out of those 12 patients (58%), the arthritis and/or arthralgia in the joints of the hands were documented in medical records. Fifty-eight percent of individuals with CTS were classified as ASS patients, whereas CTS was significantly overrepresented in the ASS group (30%) as compared to other IIMs (10%). CTS was mainly associated with the presence of anti-synthetase antibodies (58%) or anti-Mi-2β antibodies (25%).

### 3.3 MSA and MAA in relation to the clinical subtypes of IIM

The presence of myositis-specific and myositis-associated autoantibodies (MAAs) in the study cohort is shown in Table 2, and the clinical disease features related to MSAs are shown in Table 3.

**Table 2:** Distribution of myositis-specific (MSA) and myositis-associated autoantibodies (MAA) in a cohort of 70 patients with idiopathic inflammatory myopathies.

Autoantibodies	Patients, *n* (%)
MSA	
Anti-Jo-1	13 (18.6%)
Anti-PL-7	9 (12.9%)
Anti-PL-12	5 (7.1%)
Anti-OJ	1 (1.4%)
Anti-Mi-2α	4 (5.7%)
Anti-Mi-2β	5 (7.1%)
Anti-SAE	2 (2.9%)
Anti-MDA-5	1 (1.4%)
Anti-NXP-2	4 (5.7%)
Anti-TIF1γ	3 (4.3%)
Anti-SRP	6 (8.6%)
Anti-HMGCR	4 (5.7%)
MAA	
Anti-SSA-52	22 (31.4%)
Anti-SSA-60	5 (7.1%)
Anti-Pm/Scl-75	6 (8.6%)
Anti-Pm/Scl-100	7 (10%)
Anti-Ku	3 (4.3%)
Anti-RNP	2 (2.9%)
Anti-cN-1A	2 (2.9%)

**Table 3:** Clinical features of myositis according to the presence of myositis-specific and -associated antibodies in a cohort of 70 patients with idiopathic inflammatory myopathies.

Autoantibodies	Patients, *n*	Median age (IQR)	Muscle symptoms	Raynaud syndrome	Arthralgia/ arthritis	Pulmonary involvement	Dysphagia	Cancer diagnosis
Anti-Jo-1	13	46 (34–68)	11 (85%)	6 (46%)	12 (92%)	13 (100%)	2 (15%)	0
Anti-PL-7	9	55 (41–71)	5 (56%)	1 (11%)	3 (33%)	6 (67%)	3 (33%)	0
Anti-PL-12	5	38 (33–76)	0	2 (40%)	2 (40%)	5 (100%)	1 (20%)	1 (20%)
Anti-OJ	1	34	0	0	1 (100%)	1 (100%)	0	0
Anti-Mi-2α	4	64 (31–76)	3 (75%)	4 (100%)	3 (75%)	0	0	0
Anti-Mi-2β ^a^	5	54 (38–77)	5 (100%)	2 (40%)	2 (40%)	0	2 (40%)	1 (20%)
Anti-SAE	2	61 (52–69)	2 (100%)	0	1 (50%)	2 (100%)	1 (50%)	1 (50%)
Anti-MDA-5	1	54	0	0	1 (100%)	1 (100%)	0	0
Anti-NXP-2	4	47 (35–62)	4 (100%)	1 (25%)	2 (50%)	0	3 (75%)	2 (50%)
Anti-TIF1γ	3	84 (57–87)	3 (100%)	0	0	0	1 (33%)	0
Anti-SRP	6	64 (33–81)	6 (100%)	3 (50%)	1 (17%)	2 (33%)	3 (50%)	3 (50%)
Anti-HMGCR	4	70 (60–76)	3 (75%)	0	1 (25%)	0	3 (75%)	0
Anti-Pm/Scl-75 ^b^	6	38 (30–61)	5 (83%)	4 (67%)	3 (50%)	4 (67%)	0	0
Anti-Pm/Scl-100	7	47 (34–60)	6 (86%)	5 (71%)	3 (43%)	5 (71%)	1 (14%)	0
Anti-Ku	3	34 (25–87)	2 (67%)	1 (33%)	1 (33%)	1 (33%)	2 (67%)	0
Anti-RNP	2	68 (62–73)	2 (100%)	2 (100%)	2 (100%)	1 (50%)	0	0
Anti-cN-1A	2	75 (69–80)	2 (100%)	1 (50%)	2 (100%)	1 (50%)	0	0

The number and the percentage of patients presenting respective clinical features within the MSA/MAA antibody group are indicated.

^a^ One patient had both positive anti-Mi-2α and anti-Mi-2β.

^b^ Three patients were both positive for anti-Pm/Scl-75 and anti-Pm/Scl-100.

A defined subset of IIM—IMNM—was diagnosed and biopsy- verified in 16% of the cohort (*n* = 11), whereas 36% (*n* = 4) of patients had positive anti-HMGCR antibodies and 54% (*n* = 6) displayed anti-SRP antibodies. Anti-HMGCR-positive myositis was associated with a history of statin treatment in 75% of cases. Interestingly, pulmonary involvement was seen in 18% of patients, whereas esophagus involvement was relatively common—55% in the whole IMNM group and 75% of anti-HMGCR-positive patients.

Cancer-associated myopathy (CAM) was identified in 13% of patients (*n* = 9). Most of the patients (89%) presented positive MSA: antibodies against SRP (*n* = 3), NXP2 (*n* = 2), Mi-2β (*n* = 1), SAE (*n* = 1), and PL-12 (*n* = 1) were detected. The most prevalent (44%) was gynecological malignancy in terms of ovarian or uterus cancer (*n* = 4) followed by breast cancer (*n* = 2), colon cancer (*n* = 1), spread thymoma (*n* = 1), and spread malign lentigo of the skin (*n* = 1). Importantly, patients with CAM displayed new skin symptoms in 89% of cases.

The classification criteria for definite ASS, according to Connor et al. (5) were fulfilled in 33% (*n* = 23) of patients in our IIM cohort. Anti-Jo-1, the most common MSA in ASS patients, was present in 56% of patients, followed by anti-PL-7 in 37% and anti-PL-12 in 22% of cases. Anti-Jo-1 was also the most prevalent MSA in our IIM cohort, detected in a total of 19% of patients (*n* = 13), whereas the most common MAA was anti-Ro/SSA52 in 31% of patients (*n* = 22).

Definite IBM according to the classification criteria by Griggs et al. (6) was confirmed in three patients, and two of them had positive anti-cN1A antibodies.

The presence of MSA was relatively monospecific, and the simultaneous occurrence of more than one MSA was seen in 7% of patients (*n* = 5). The coincidence of anti-PL-7 with another MSA was most common and was detected together with anti-Jo-1 (*n* = 1), anti-PL-12 (*n* = 1), anti-Jo-1 plus anti-OJ (*n* = 1), and anti-HMGCR (*n* = 1). One patient had both positive anti-Mi-2α and anti-Mi-2β antibodies. In terms of MAA, 10% of patients (*n* = 7) had more than one antibody. A coincidence of anti-Pm/Scl-75 with anti-Pm/Scl-100 was seen alone (*n* = 1) or together with anti-RNP (*n* = 1) or anti-Ro/SSA52 (*n* = 1). A combination of antibodies against Ro/SSA52 together with anti-cN-1A (*n* = 1) and anti-Pm/Scl-100 (*n* = 2) was detected, as well as a combination of anti-Scl-75 and anti-Ku (*n* = 1).

### 3.4 MSA and MAA in relation to pulmonary involvement

In our cohort, 47% (*n* = 33) of the patients in the whole study group, 86% (*n* = 19) of patients with anti-Ro/SSA52 positivity, and 100% (*n* = 13) with anti-Jo-1 had lung involvement. Of the patients with pulmonary involvement, the majority (*n* = 21) were diagnosed with ASS, whereas 24% (*n* = 8) were classified as having DM and 12% (*n* = 4) as having PM.

From the whole cohort, 38% of patients were ever-smokers, but only 4.4% smoked at the time of diagnosis. No significant differences were seen between the groups, 42% (*n* = 14) of the patients with lung involvement and 32% (*n* = 12) of the patients without lung involvement were ever-smokers.

**Figure 2:**
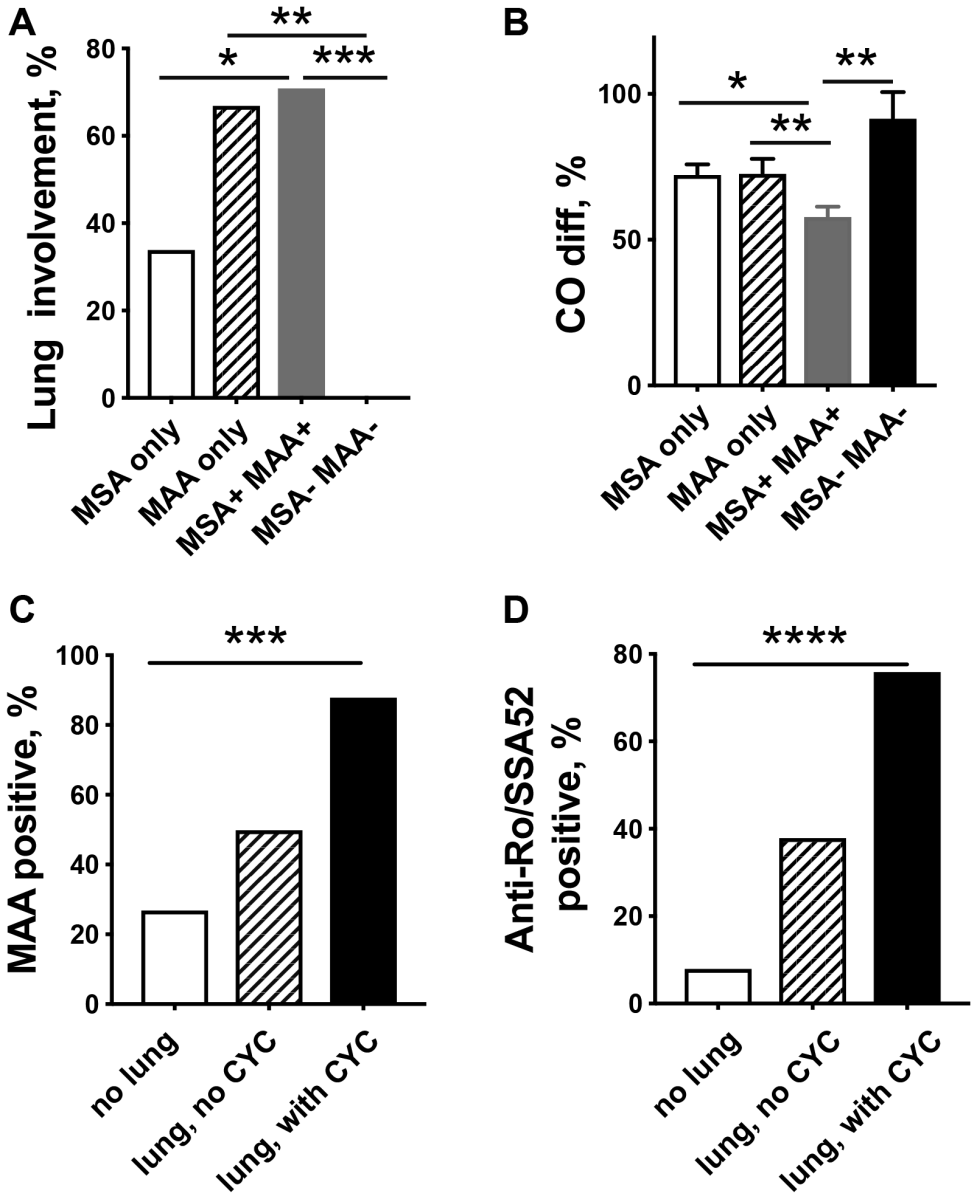
Relationship between myositis-specific antibodies (MSAs), myositis-associated antibodies (MAAs), and pulmonary involvement in patients with idiopathic inflammatory myopathies. **(A)** Lung involvement is significantly more common in patients presenting MAA only or MAA/MSA as compared to IIM patients with only MSA. **(B)** Patients with the MSA/MAA combination have significantly decreased CO-diffusion capacity compared to other patient groups. MAA **(C)** and especially anti-Ro/SSA52 positivity **(D)** are related to more severe lung involvement in need of cyclophosphamide (CYC) treatment. * *p* < 0.05, ** *p* < 0.01, ***p < 0.001, **** *p* < 0.0001.

Patients with the presence of MSA together with MAA and patients with only MAA had a significantly higher incidence of lung disease compared to patients with MSA only (Figure 2A). None of the patients suffered from lung disease due to IIM if only antibodies against Mi-2α, Mi-2β, NXP2, HMGCR, and TIF1γ were present or no MSA/MAA were detected. In addition, significantly decreased CO-diffusion capacity was registered in patients who presented both MSA and MAA compared with other patient groups (Figure 2B).

To estimate the need for immunosuppression during the disease course for each patient, we calculated the treatment score. Our results show that patients with lung involvement required significantly more intense treatment (average treatment weight score 5.9 vs. 1.8) and higher doses of GCs (calculated mean equivalent dose of prednisolone at diagnosis: 108 mg vs. 42 mg).

To identify special characteristics in the most severe disease in patients with lung involvement, we divided the 33 patients into 2 groups: the patients who were in need of treatment with CYC (*n* = 17) and a non-CYC group (*n* = 16). Patients in the CYC group had a lower mean CO-diffusion capacity (53% vs. 68%), were treated with higher doses of prednisolone at diagnosis (183 mg vs. 28 mg), and had a higher average total treatment weight score (8.4 vs. 3.4) compared to the non-CYC group, respectively.

Interestingly, patients in the CYC group had a significantly higher frequency of MAA (88% vs. 50%, *p* = 0.004) (Figure 2C) and were significantly more often anti-Ro/SSA52 positive (76% vs. 38%, *p* < 0.001) (Figure 2D), whereas no differences were seen in the presence of MSA (82% vs. 81%) as compared to patients in the non-CYC group.

## 4 Discussion

IIM is a diverse heterogenous disease consisting of various subgroups, each exhibiting distinct clinical manifestations. Notably, different phenotypes of IIM are linked to specific myositis autoantibodies, making these autoantibodies valuable biomarkers for disease diagnosis, subtyping, and prognosis prediction. In this study, we investigated the relationship between the presence of myositis autoantibodies and various disease manifestations in a cohort of 70 well-characterized IIM patients from a single center. Our results revealed that the presence of MAAs, particularly anti-Ro/SSA52 antibodies, is associated with a higher risk of developing interstitial lung diseases (ILDs) in myositis patients. Furthermore, patients who tested positive for both MSA and MAA demonstrated lower diffusing capacity for carbon monoxide compared to those who were positive for either MSA or MAA alone.

Our findings are consistent with previous research. In a prospective study involving 315 patients diagnosed with IIM, the presence of anti-Ro/SSA52 antibodies was associated with the progression of ILDs. Among patients in the ASS group who tested negative for anti-Ro/SSA52 antibodies, there was a higher frequency of association with alveolitis, and those patients responded well to immunosuppressive therapy. In contrast, the anti-Ro/SSA52-positive group exhibited more fibrosis on high-resolution computed tomography scans. The authors concluded that the coexistence of anti-Ro/SSA52 and anti-Jo-1 antibodies could serve as a valuable predictor for the development of a more severe and advanced form of ILD in patients with IIM. Such patients may require a more aggressive therapeutic approach, as indicated by the findings from the study (8). Additionally, La Corte et al. demonstrated that ASS patients with associated anti-Ro/SSA52 antibodies were predisposed to the development of a more severe ILD (9). Our data also showed a progressive increase in the frequency of anti-Ro/SSA52 antibodies, rising from approximately 7% in patients without pulmonary involvement to 40% in patients with pulmonary involvement who did not require CYC treatment. In patients with pulmonary involvement who are in need of CYC treatment, the frequency of anti-Ro/SSA52 antibodies reached approximately 80%.

Anti-Ro/SSA52 antibodies consistently demonstrate associations with ILDs and declining lung function in various rheumatic conditions, underscoring their clinical significance in these contexts. In patients with mixed connective tissue disease (MCTD), research has revealed an association between anti-Ro/SSA52 antibodies and lung fibrosis. Among a cohort of 113 MCTD patients, 34% were confirmed to have lung fibrosis using HRCT scans. Interestingly, 50% of MCTD patients with lung fibrosis tested positive for anti-Ro/SSA52 antibodies, while only 19% of those without lung fibrosis presented anti-Ro/SSA52 (10). In individuals with SSc, ILD remains the leading cause of mortality. Remarkably, the presence of anti-Ro/SSA52 antibodies, as opposed to anti-Scl-70 antibodies, has been significantly linked to progressive ILD and the gradual loss of lung function. The rate of lung function decline demonstrated a linear increase with rising levels of anti-Ro/SSA52 antibodies (11). Consistent with these findings, another study encompassing connective tissue diseases reported that anti-Ro/SSA52 positivity is associated with poorer survival rates in SSc patients (12). Furthermore, anti-Ro/SSA52 antibodies have been identified as a risk factor for developing ILD in pSS. In a retrospective study involving 68 pSS patients, the presence of anti-Ro/SSA52 antibodies was significantly associated with a higher incidence of ILD compared to those without these antibodies (13).

The involvement of autoantibodies in the pathogenesis of rheumatic diseases is suggested, as seen in ACPA-mediated bone loss in RA (14) and ANCA in vasculitis (15). The direct contribution of anti-Ro/SSA52 antibodies to the disease’s development is very clear in congenital heart block in neonatal lupus syndrome. The transplacental transfer of maternal anti-Ro/SSA52 is associated with irreversible damage to the fetal cardiac conduction system. Several studies have demonstrated direct effects of anti-Ro/SSA52 antibodies on cardiocyte function, possibly due to cross-reactivity. Anti-Ro/SSA52 antibodies that target p200 were found to directly interact with cardiomyocytes and disrupt calcium homeostasis (16). Human affinity-purified anti-Ro-52-positive sera were shown to induce cardiac conduction disorders in young rabbit hearts, similar to those observed in neonatal lupus (17). Additionally, immunizing female mice with recombinant SSA/Ro-52 KD protein resulted in atrioventricular conduction defects in their offspring (18). However, it remains largely unclear whether anti-Ro/SSA52 antibodies are merely biomarkers for ILD or play a causative role in the mechanism of ILD. Nevertheless, a recent report indicates that the majority of SSc-ILD patients who tested positive for anti-Ro/SSA52 exhibited a significant enrichment of anti-Ro/SSA52 antibodies in their BAL fluid, with a ratio exceeding 50 times (11), suggesting a potential pathological role of Ro-52 antibodies in pulmonary pathology. Further research is, however, needed to fully understand this phenomenon.

In our study, we made a noteworthy observation regarding the prevalence of CTS among patients with ASS. We found that CTS was significantly overrepresented in ASS patients and was strongly associated with the presence of anti-synthetase antibodies or anti-Mi-2β antibodies. CTS is a relatively common condition in the realm of rheumatic diseases, including RA (19), PsA (20), systemic lupus erythematosus (SLE) (21), and SSc (22). For instance, previous studies have reported the electrophysiological frequencies of CTS at 13.2, 15.4, and 3.5% in the RA, PsA, and control groups, respectively (20). Remarkably, the frequency of CTS in our IIM cohort was even higher, at 17%, reaching what has been reported for RA and PsA. Notably, within the ASS subgroup, the incidence of CTS was particularly striking, exceeding 50%. This finding suggests that there may be unique underlying mechanisms or factors specific to ASS that contribute to an increased risk of CTS compared to other rheumatic conditions. The joint inflammation in the wrists could be one contributing factor since arthritis/arthralgia was documented in the medical records of more than half of patients with CTS. Since our study had a retrospective nature, some information about the presence of arthritis could be missed. Our study adds a novel dimension to the understanding of CTS within the context of myositis, highlighting the need for further investigation into the pathophysiological links between myositis, particularly ASS, and CTS. These findings underscore the complexity and heterogeneity of rheumatic diseases and warrant future research to elucidate the underlying mechanisms driving this pronounced association.

While our investigation has provided valuable insights into the clinical correlations of myositis autoantibodies, it is essential to acknowledge certain limitations in our study. Due to the retrospective nature of the study, it is possible that some information might be missing or incompletely documented in medical databases. Recognizing the relatively limited number of participants, the role of some infrequent autoantibodies cannot be evaluated. Additionally, since the primary focus of our research was unraveling the clinical associations of myositis autoantibodies, we excluded the patients for whom the myositis autoantibody analysis was not available. This led to the exclusion of individuals with confirmed myositis diagnoses and included mostly patients before line blot analysis became commercially available in our laboratory. However, this exclusion was not intended to introduce bias into our study outcome. Instead, it was a deliberate choice aimed at enhancing the better characterization of our patient cohort.

In summary, our single-center retrospective study involving 70 well-characterized IIM patients reveals that the presence of MAA autoantibodies, particularly anti-Ro/SSA52 in conjunction with MSA autoantibodies, is associated with more severe ILD in myositis patients. This association necessitates more aggressive immunosuppressive treatments. To predict disease severity and plan treatment effectively, a comprehensive myositis autoantibody profile is essential.

## Data availability statement

The raw data supporting the conclusions of this article will be made available by the authors, without undue reservation.

## Ethics statement

The studies involving humans were approved by regional Swedish Ethical Review Board, University of Gothenburg (Dnr 449–12). The studies were conducted in accordance with the local legislation and institutional requirements. Written informed consent for participation was not required from the participants or the participants’ legal guardians/next of kin because the study was a retrospective analysis of patients medical records.

## Author contributions

JM: Data curation, Formal analysis, Investigation, Methodology, Writing – original draft. BH: Data curation, Investigation, Writing – review & editing. TJ: Formal analysis, Investigation, Supervision, Validation, Visualization, Writing – review & editing, Funding acquisition. RP: Conceptualization, Formal analysis, Funding acquisition, Investigation, Methodology, Project administration, Resources, Supervision, Validation, Writing – original draft, Writing – review & editing.

## References

[1] Lundberg IE Miller FW Tjärnlund A Bottai M. Diagnosis and classification of idiopathic inflammatory myopathies. J Intern Med. (2016) 280:39–51. doi: 10.1111/joim.12524, PMID: 27320359 PMC5021058

[2] Lundberg IE Fujimoto M Vencovsky J Aggarwal R Holmqvist M Christopher-Stine L. Idiopathic inflammatory myopathies. Nat Rev Dis Primers. (2021) 7:86. doi: 10.1038/s41572-021-00321-x 34857798

[3] Bohan A Peter JB. Polymyositis and dermatomyositis (second of two parts). N Engl J Med. (1975) 292:403–7. doi: 10.1056/NEJM197502202920807 1089199

[4] Bohan A Peter JB. Polymyositis and dermatomyositis (first of two parts). N Engl J Med. (1975) 292:344–7. doi: 10.1056/NEJM197502132920706 1090839

[5] Connors GR Christopher-Stine L Oddis CV Danoff SK. Interstitial lung disease associated with the idiopathic inflammatory myopathies: what progress has been made in the past 35 years? Chest. (2010) 138:1464–74. doi: 10.1378/chest.10-0180 21138882

[6] Griggs RC Askanas V DiMauro S Engel A Karpati G Mendell JR. Inclusion body myositis and myopathies. Ann Neurol. (1995) 38:705–13. doi: 10.1002/ana.410380504 7486861

[7] Mitander A Fei Y Trysberg E Mohammad M Hu Z Sakiniene E. Complement consumption in systemic lupus erythematosus leads to decreased Opsonophagocytosis in vitro. J Rheumatol. (2018) 45:1557–64. doi: 10.3899/jrheum.171325, PMID: 30173146

[8] Váncsa A Csípő I Németh J Dévényi K Gergely L Dankó K. Characteristics of interstitial lung disease in SS-A positive/Jo-1 positive inflammatory myopathy patients. Rheumatol Int. (2009) 29:989–94. doi: 10.1007/s00296-009-0884-9, PMID: 19266202

[9] la Corte R Lo Mo naco A Locaputo A Dolzani F Trotta F. In patients with antisynthetase syndrome the occurrence of anti-Ro/SSA antibodies causes a more severe interstitial lung disease. Autoimmunity. (2006) 39:249–53. doi: 10.1080/08916930600623791, PMID: 16769659

[10] Gunnarsson R el-Hage F Aaløkken TM Reiseter S Lund MB Garen T. Associations between anti-Ro52 antibodies and lung fibrosis in mixed connective tissue disease. Rheumatology. (2016) 55:103–8. doi: 10.1093/rheumatology/kev300, PMID: 26320136

[11] Hamberg V Sohrabian A Volkmann ER Wildt M Löfdahl A Wuttge DM. Anti-Ro52 positivity is associated with progressive interstitial lung disease in systemic sclerosis-an exploratory study. Arthritis Res Ther. (2023) 25:162. doi: 10.1186/s13075-023-03141-4, PMID: 37667402 PMC10476305

[12] Decker P Moulinet T Lopez B Dubucquoi S Bonnotte B Lakomy D. Clinical significance of anti-Ro52 (TRIM21) antibodies in adult patients with connective tissue diseases. Eur J Intern Med. (2021) 91:45–52. doi: 10.1016/j.ejim.2021.04.020, PMID: 33972152

[13] Buvry C Cassagnes L Tekath M Artigues M Pereira B Rieu V. Anti-Ro52 antibodies are a risk factor for interstitial lung disease in primary Sjogren syndrome. Respir Med. (2020) 163:105895. doi: 10.1016/j.rmed.2020.105895, PMID: 32056839

[14] Krishnamurthy A Joshua V Haj Hensvold A Jin T Sun M Vivar N. Identification of a novel chemokine-dependent molecular mechanism underlying rheumatoid arthritis-associated autoantibody-mediated bone loss. Ann Rheum Dis. (2016) 75:721–9. doi: 10.1136/annrheumdis-2015-208093, PMID: 26612338 PMC4819614

[15] Brouwer E Huitema MG Klok PA de Weerd H Tervaert JW Weening JJ. Antimyeloperoxidase-associated proliferative glomerulonephritis: an animal model. J Exp Med. (1993) 177:905–14. doi: 10.1084/jem.177.4.905, PMID: 8384653 PMC2190976

[16] Salomonsson S Sonesson SE Ottosson L Muhallab S Olsson T Sunnerhagen M. Ro/SSA autoantibodies directly bind cardiomyocytes, disturb calcium homeostasis, and mediate congenital heart block. J Exp Med. (2005) 201:11–7. doi: 10.1084/jem.20041859 15630133 PMC2212767

[17] Garcia S Nascimento JH Bonfa E Levy R Oliveira SF Tavares AV. Cellular mechanism of the conduction abnormalities induced by serum from anti-Ro/SSA-positive patients in rabbit hearts. J Clin Invest. (1994) 93:718–24. doi: 10.1172/JCI117025 8113406 PMC293909

[18] Stolowich NJ Frolov A Atshaves B Murphy EJ Jolly CA Billheimer JT. The sterol carrier protein-2 fatty acid binding site: an NMR, circular dichroic, and fluorescence spectroscopic determination. Biochemistry. (1997) 36:1719–29. doi: 10.1021/bi962317a, PMID: 9048555

[19] Karadag O Kalyoncu U Akdogan A Karadag YS Bilgen SA Ozbakır S. Sonographic assessment of carpal tunnel syndrome in rheumatoid arthritis: prevalence and correlation with disease activity. Rheumatol Int. (2012) 32:2313–9. doi: 10.1007/s00296-011-1957-0, PMID: 21607558

[20] Kaya Subaşı P Güler T Yurdakul FG Ataman Ş Bodur H. Carpal tunnel syndrome in patients with rheumatoid arthritis and psoriatic arthritis: an electrophysiological and ultrasonographic study. Rheumatol Int. (2021) 41:361–8. doi: 10.1007/s00296-020-04745-8, PMID: 33185703

[21] Sivri A Hasçelik Z Celiker R Başgöze O. Early detection of neurological involvement in systemic lupus erythematosus patients. Electromyogr Clin Neurophysiol. (1995) 35:195–9. PMID: 7555923

[22] Machet L Vaillant L Machet MC Esteve E Muller C Khallouf R. Carpal tunnel syndrome and systemic sclerosis. Dermatology. (1992) 185:101–3. doi: 10.1159/000247422 1421620

